# Temozolomide in paediatric high-grade glioma: a key for combination therapy?

**DOI:** 10.1038/sj.bjc.6601997

**Published:** 2004-07-20

**Authors:** A C Verschuur, J Grill, A Lelouch-Tubiana, D Couanet, C Kalifa, G Vassal

**Affiliations:** 1 Department of Paediatric Oncology, Institut Gustave Roussy, 39 rue Camille Desmoulins, 94805 Villejuif Cedex, France; 2 Department of Pathology, Hôpital Necker-Enfants Malades, 149 rue de Sèvres, 75015 Paris, France; 3 Department of Radiology, Institut Gustave Roussy, 39 rue Camille Desmoulins, 94805 Villejuif Cedex, France; 4 Department of Paediatric Oncology, Academic Medical Centre, University of Amsterdam, Emma Childrens' Hospital AMC, F8-243, PO Box 22700, 1100 DE Amsterdam, The Netherlands

**Keywords:** temozolomide, high-grade glioma, children

## Abstract

This report describes a single-centre study with temozolomide (TMZ) (200 mg m^−2^ day^−1^ × 5 per cycle of 28 days) in children with (recurrent) high-grade glioma. Magnetic resonance imaging was performed every two cycles. In all, 20 patients were treated between 1998 and 2001 after the UKCCSG/SFOP TMZ phase II trial. All patients had measurable disease. Totally, 15 patients had a relapse after surgery±radiotherapy±chemotherapy. Overall, five patients received TMZ after surgery or biopsy, awaiting radiotherapy. There were one clinically malignant grade II glioma, 11 grade III and eight grade IV gliomas. Seven tumours had oligodendroglial features. Mean age at start of TMZ was 12.0 years (range 3–20.5 years). In total, eight patients had >8 cycles (range 3–30). One VGPR (currently in CR after surgery), three PRs (with a PFS of 4, 4 and 11 months, respectively) and one MR (PFS 14 months) were observed. Three out of five responses occurred after >4 courses. The overall response rate was 20%. Median progression-free survival (PFS) was 2.0 months (range 3 weeks–34^+^ months). PFS rate was 20% after 6 months. Median overall survival (OS) was 10 months. Nine patients showed a clinical improvement. Three patients vomitted shortly after TMZ administration, eight patients (13 cycles) experienced grade III/IV thrombocytopenia, occurring predominantly during the fourth week of the first two cycles. Five patients experienced neutropenia, and three patients febrile neutropenia. TMZ is a well-tolerated ambulatory treatment for children with malignant glial tumours. This drug warrants further study in these highly chemoresistant tumours and should be studied either as upfront therapy or in combination therapy.

Temozolomide (TMZ) is an oral methylating agent that belongs to the *imidazotetrazine* class of drugs. It is metabolised into monomethyl triazeno imidazole carboxamide (MTIC) (Stevens *et al*, 1987), which is also the cytotoxic metabolite of dimethyl triazeno imidazole carboxamide (DTIC, dacarbazine). The cytotoxic effect of MTIC is caused by alkylation of deoxyguanosine nucleotides, mostly at the O^6^ position, leading to DNA mismatch (G–T match instead of G–C match) (Catapano *et al*, 1987), DNA strand breaks and subsequently apoptosis (Tentori *et al*, 1997).

Several phase I studies with TMZ single drug have been published in adults and children with solid tumours (Newlands *et al*, 1992; O'Reilly *et al*, 1993; Dhodapkar *et al*, 1997; Brock *et al*, 1998; Estlin *et al*, 1998; Nicholson *et al*, 1998; Brada *et al*, 1999), showing haematological dose-limiting toxicity (DLT). Maximally tolerated doses (MTD) were between 150 and 250 mg m^−2^ day^−1^ when using the once daily oral administration on 5 consecutive days every 4 weeks, depending on whether or not patients had been previously treated with nitrosureas (Dhodapkar *et al*, 1997) or craniospinal irradiation (CSI) (Nicholson *et al*, 1998). In children, the MTD is 180 mg m^−2^ day^−1^ for patients with previous CSI (Nicholson *et al*, 1998) and 200–215 mg m^−2^ day^−1^, respectively for patients without prior CSI (Estlin *et al*, 1998; Nicholson *et al*, 1998, respectively). The reports of phase II trials of TMZ using comparable administration regimens of 150–200 mg m^−2^ day^−1^ × 5 every 28 days in adults with recurrent or progressive grade III or grade IV (oligodendro-) glioma showed objective response rates varying from 8, 11, 23, 35 and 44%, respectively (Brada *et al*, 2001; Bower *et al*, 1997; Brandes *et al*, 2001; Yung *et al*, 1999; Chinot *et al*, 2001, respectively). A phase II clinical trial by the UKCCSG New Agents Group/SFOP ‘Groupe de Pharmacologie’ with this regimen of TMZ in paediatric patients with recurrent or progressive high-grade glioma showed a lower response rate of 12% for supratentorial high-grade glioma and 6% for brainstem glioma, respectively (Lashford *et al*, 2002). In this study, the response rate was evaluated after two cycles of TMZ. Despite the low response rate, several patients improved clinically suggesting that TMZ is a good palliative treatment in patients with malignant glioma. We report here a single institution experience using TMZ in children with (recurrent) grade III or IV glioma, focussing on clinical improvement, response rate, progression-free survival (PFS) and toxicity.

## MATERIALS AND METHODS

### Patients

The inclusion period of our study (from May 1998 to February 2001) started after the accomplishment of the UKCCSG/SFOP New Agents Group TMZ phase II trial. Patients of 1–21 years of age were eligible with grade III or grade IV glioma, including high-grade oligodendroglioma. Patients with brainstem glioma were excluded. Histology was evaluated centrally.

In all, 15 patients were included in the study population while showing a recurrence or progression after neurosurgery±radiotherapy±chemotherapy. Prior chemotherapy within the last 4 weeks was an exclusion criteria. Five patients received TMZ at initial diagnosis while awaiting radiotherapy after having had a resection or a biopsy. Patients' characteristics are mentioned in Table 1

**Table 1:** Patients' characteristics and outcome of the study population

**Patient no.**	**Histology nominal**	**Histology grade**	**Therapy before TMZ**	**Status before relapse**	**Metastasis**	**Age at start TMZ (years)**	**Corticosteroids?**	**Clinical improvement**	**Response**	**PFS (months)**
1	Oligodendroglioma	II	CH+RT	PD	Y	6.9	Y	(See text)	VGPR	34+
2	Glioblastoma	IV	SU+RT+CH	VGPR I	N	9.9	N	Hemiplegia –	PR	11
								seizures –		
3^a^	Malignant glioma	IV	SU	PR I	N	15.8	Y	Corticosteroids –	PR	4
4	Anaplastic oligodendroglioma	III	CH	PD	Y	9.5	N	Hemiplegia –seizures –	MR	14
								vomiting –		
5	Anaplastic glioma	IV	SU+RT+CH	PR I	Y	8.1	Y	No	PD	0.75
6	Gemistiocytic astrocytoma	III	SU+CH+SU+SU+RT	PR 2	Y	3	N	No	PD	0.75
7	Anaplastic oligodendroglioma	III	SU+CH+CH+RT+SU	CR 3	N	5.7	N	No	PD	2
8	Malignant glioma	IV	SU+RT	PR 2	Y	18.2	N	No	PD	1.5
9	Anaplastic oligodendroglioma	III	SU+RT+CH	PR 1	N	14.7	N	No	PD	1.5
10	Malignant glioma	IV	SU+RT	PR 2	N	18.9	Y	No	PD	2
11^a^	Anaplastic ganglioglioma	III	SU	PR 1	N	9.5	N	No	PD	1
12^a^	Astrocytoma	III			N	9.5	Y	No	PD	2
13	Medullary glioblastoma	IV	SU+SU+RT	CR 5	N	18.7	N	No	PD	2
14	Gemistocytic astrocytoma	III	SU+RT	PR	Y	17	Y	Briefly improved swallowing	PD	2
15^a^	Malignant glioma	IV	SU	PR	N	6.9	Y	Hemiplegia –	PD	2.5
								diplopia –		
								corticosteroids –		
16	Anaplastic oligodendroglioma	III	SU+RT+CH	CR	N	13.9	N	No	PR	4
17^a^	Anaplastic oligodendroglioma	III			N	8.8	N	Hemiplegia –	PD	3.5
18	Glioblastoma	IV	SU+RT	PR	Y	20.5	N	No	PD	2
19	Oligodendrocytoma	III	SU+RT+CH+CH+RT+CH+RTCHIR	PR 5	N	10.1	Y	Hemiplegia –vomiting –	PD	2
								corticosteroids –		
20	Astroblastoma	III	CH+CH+SU+CH+RT	PR 2	N	14.1	Y	Hemiplegia –	PD	7
								vomiting –		
								corticosteroids –		

a Patients were treated prior to radiotherapy. RT=radiotherapy; SU=surgery; Ch=chemotherapy; VGPR=very good partial response; PR=partial response; MR=minor response; PD=progressive disease. A – sign in the ‘clinical improvement’ column means that the symptom disappeared or corticosteroid therapy was weaned.

. All patients had measurable disease.

### Drug administration and assessment of response and toxicity

TMZ (Temodal®, 200 mg m^−2^ day^−1^ orally) was administered once a day on 5 consecutive days per cycle of 28 days. Ingestion occurred in the morning 30 min before breakfast. In case of grade I (or more) nausea or vomiting, ondansetron was administered 30 min before the administration of TMZ. The dose of TMZ was decreased by 20% for the subsequent cycle in case of repeated or prolonged (>7 days) grade III/IV haematological toxicity. This dose decrement could be repeated for subsequent cycles.

Magnetic resonance imaging was performed prior to the start of TMZ treatment and was repeated every two cycles. All imaging was reviewed to determine any response. Response criteria were according to WHO (WHO, 1979), using the product of the two maximal diameters of the tumour. Best response was determined at any evaluation moment during the treatment period. Treatment was continued in case of minor, partial or complete response or stable disease. Progression-free survival was defined as the interval between the first day of the first cycle of TMZ and the occurrence of tumour progression.

Toxicity was assessed according to the NCI-CTC. Full blood count was analysed once a week and once to twice a week during the third and fourth week of each cycle. Thrombocyte transfusions were administered when platelet count dropped below 50 × 10^9^ l^−1^.

## RESULTS

Totally, 20 patients were treated between May 1998 and May 2001. Follow-up was until May 2002. Patients' characteristics are shown in Table 1. Seven patients had metastatic disease and nine patients were on corticosteroid therapy when starting TMZ treatment. Eight patients had a relapse after surgery+radiotherapy+chemotherapy, five patients after surgery+radiotherapy, one patient after chemotherapy+radiotherapy, and one patient after chemotherapy alone. Three patients received TMZ after incomplete resection and two patients after having had a biopsy only, awaiting radiotherapy. Of these 18 patients, response to prior therapy was CR for three patients (one CR1, one CR3, one CR5), one VGPR, 12 PR (seven PR1, four PR2, one PR5) and two PD. Mean age at start of TMZ was 12.0 year (range 3–20.5 year). Median number of cycles was 2.0 (range 1–30^+^).

**Figure 1:**
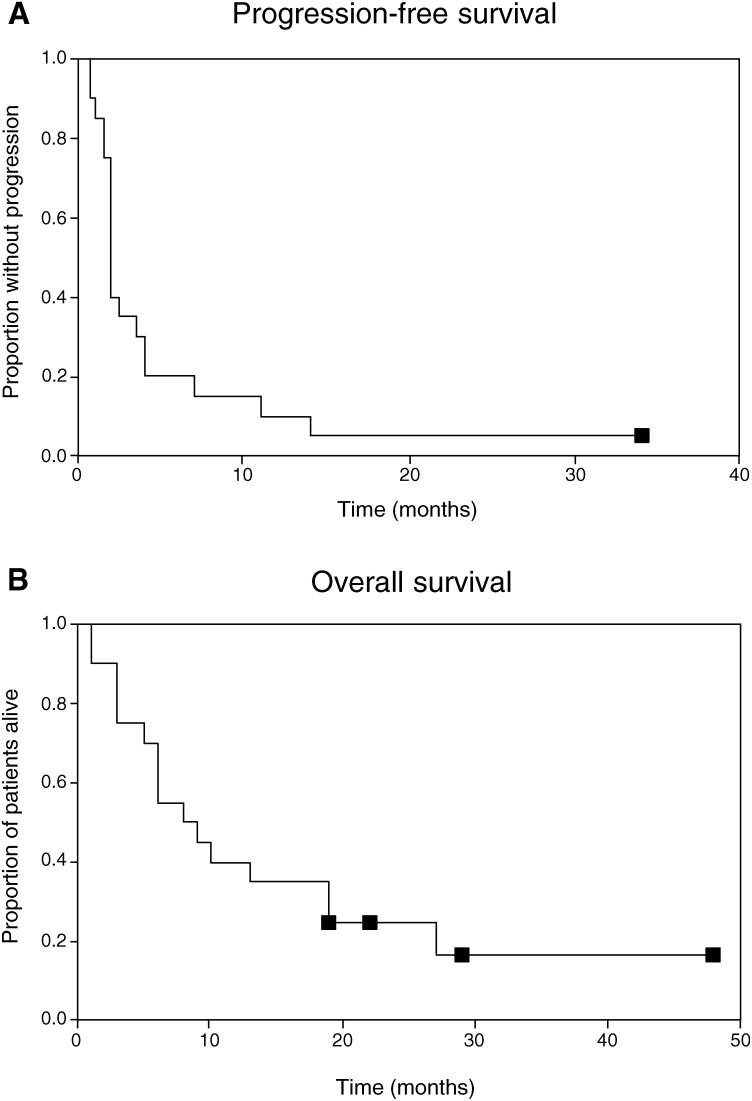
Progression-free survival (**A**) and overall survival (**B**) of the total study population as determined from the start of temozolomide treatment for each patient. ▪ Symbols represent censored observations. Median PFS was 2 months. Median OS was 10 months.

We observed one VGPR (currently in CR after surgery), and three PRs (with a PFS of 4, 4 and 11 months, respectively) and one MR (PFS 14 months). Three of the five responses were observed in oligodendroglioma. Three out of five responses occurred after >4 courses. Median PFS was 2.0 months (range 3 weeks–34 months^+^). The overall response rate (CR+PR) was 20% (95% CI 2–38%). PFS rate was 20% after 6 months (Figure 1A

). Median overall survival was 10 months (Figure 1B). Nine patients showed a clinical improvement (Table 1); 50% of the patients on corticosteroid therapy could reduce or stop corticosteroids.

Two patients experienced a clinical deterioration during the first cycle of TMZ, while they improved clinically and radiologically during the subsequent courses.

Three patients vomitted shortly after TMZ administration. Eight patients experienced grade III/IV thrombocytopenia: platelet nadir could not be determined since the patients received platelet transfusions (total of 23 transfusions in 13 cycles) when platelet counts dropped below 50 × 10^9^ l^−1^. A large majority of thrombocytopenic events occurred during the fourth week of the TMZ cycles. Seven out of eight patients experienced thrombocytopenia during the first two cycles. Five patients (one cycle each) developed neutropenia (four of which during the first cycle), and three patients febrile neutropenia. Doses of TMZ were reduced in three patients. There was no treatment-related mortality.

Of the five patients that were treated prior to radiotherapy, one patient responded (patient 3).

The patient with the best response (VGPR and currently in CR after surgery, patient 1) was initially diagnosed as having a low-grade oligodendroglioma. The tumour was inoperable, and the patient received two courses of etoposide 500 mg m^−2^ and carboplatin 800 mg m^−2^, after which he progressed. Subsequently, the patient received local irradiation with 55 Gy, after which he still progressed with even leptomeningeal dissemination. The predominant localisations of the tumour were the right temporal lobe and the thalamus. Owing to this rapid and clinically malignant evolution in 6 months, we judged that the clinical behaviour of the tumour was more compatible with a high-grade glioma. Therefore, we started TMZ treatment, leading to a PR after six courses, a VGPR after 16 cycles and subsequently a CR after surgery. At the start of TMZ treatment he presented with a left-sided haemiplegia and facial nerve palsy, coordination and behavioural abnormalities and seizures, and required 1.5 mg kg^−1^ day^−1^ of prednisolone. After initial improvement after two courses of TMZ, a clinical deterioration was observed after the fourth course which was probably due to an intratumoural haemorrhage, requiring a temporary increase in corticosteroid dose. Subsequently, his clinical condition reimproved and after eight courses of TMZ, corticosteroid therapy could be stopped, while only a minor pyramidal tract syndrome persists. He currently has normal school activities and plays basketball. Although the clinical behaviour and radiological features of the tumour were in favour of a high-grade glioma, central histological review confirmed the initial histological diagnosis of low-grade glioma.

## DISCUSSION

The 20% objective response rate as observed in our series is slightly better than the results of the British/French collaborative paediatric study group, reporting a response rate of 12 and 6% in high-grade glioma and brainstem glioma, respectively (Lashford *et al*, 2002). Our study reports the best response at any moment during TMZ treatment, while in the French/British study the response was evaluated after two cycles of TMZ. The reports of the various phase II TMZ studies performed in adults with recurrent malignant glioma using comparable dosing regimens as used in our study describe a broad spectrum of activity. The response rates of each phase II study cannot be completely compared since response was not evaluated after the same number of cycles of TMZ. Since responses may be delayed, evaluation of best response will lead to a higher response rate as compared to response evaluation after two cycles. The best results were reported in a population of adult patients with anaplastic oligodendroglioma or anaplastic oligoastrocytoma (Chinot *et al*, 2001), showing CR in 17% and PR in 27% of the patients. High-grade oligodendroglial tumours are considered as chemoresponsive (Glass *et al*, 1992) to combination regimens like PCV (procarbazine, CCNU, vincristine). In our study population, seven out of 20 tumours (35%, 95% CI 12–58%) had oligodendrial features, whose proportion may appear high. Only a small minority of glial tumours consist of oligodendroglioma (Jukich *et al*, 2001), although percentages of 33% have been described in adults in a series (Daumas-Duport *et al*, 1997). Owing to the small numbers of patients in our series, the proportion of oligodendroglioma should be interpreted with caution. Moreover, due to the small numbers it is difficult to demonstrate a more pronounced effect in the patients with oligodendroglioma (one VGPR, one PR and one MR out of seven patients) as compared to the other glial tumours (two PR out of 13 patients). Not only oligodendroglial tumours respond to TMZ, as was demonstrated by Yung *et al* (1999), who reported a response rate of 35% and disease stabilisation in 26% of their series of predominantly anaplastic astrocytoma. These response rates are higher as compared with our results that are more comparable with the response rates of 11 and 23%, respectively as observed in other phase II trials in high-grade glioma (Bower *et al*, 1997; Brandes *et al*, 2001). Even if the reported response rates of the various clinical trials are not homogeneously high, we must consider that TMZ is one of the few drugs that have shown activity as single drug in high-grade glioma. Evidence of single drug activity has to our knowledge only been demonstrated for nitrosureas, such as BCNU and CCNU (Walker *et al*, 1978, 1980; Solero *et al*, 1979) and high-dose cyclophosphamide (McCowage *et al*, 1998) that are drugs with non-neglectable toxicity.

Therefore, the reported response rates of TMZ as single drug should lead to future applications of TMZ in combination with other treatment modalities or using a different dosing schedule of TMZ as single drug. Regarding the latter, a phase I trial with an over 7-week protracted schedule of TMZ, showed an acceptable tolerance at a daily dose of 75 mg m^−2^ day^−1^ (Brock *et al*, 1998), while increasing two-fold the systemic exposure as compared to the 200 mg m^−2^ day^−1^ × 5 per 28-day schedule. A comparable ongoing phase I study in a Canadian paediatric population seems to confirm this observation (Baruchel *et al*, 2002). The results of a phase II trial with a comparable schedule in adults with relapsed malignant glioma proved not successful (Khan *et al*, 2002). However, this regimen may be suitable for combination with radiotherapy in newly diagnosed high-grade glioma. Several phase II studies with radiotherapy and TMZ are currently ongoing or have recently been completed in adults with glioblastoma multiforme (Yung *et al*, 2001). A more protracted regimen may also be attractive for the treatment of acute leukaemia, since TMZ may have some activity in this pathology as well (Seiter *et al*, 2001). Various other dosing schedules of TMZ have been studied, trying to increase the systemic exposure while not increasing the toxicity (Yung *et al*, 2001). The 300 mg m^−2^ day^−1^ × 3 per 2 week regimen is likely to increase the systemic exposure as compared with the regimen we used, and showed a response rate of 30% in primary glioma (Raymond *et al*, 2002).

Several phase I and II clinical trials combining TMZ with other cytotoxic drugs are ongoing or have been completed. The combination with carmustine (IV or polifeprosan implants) may be promising in high-grade glioma (Schold *et al*, 2000; Prados, 2001). A phase I trial with TMZ and cisplatin in adults resulted in a MTD of TMZ 200 mg m^−2^ day^−1^ for 5 days and cisplatin 75 mg m^−2^ on day 1 of a 28-day cycle (Britten *et al*, 1999). Recently, a similar study with TMZ and cisplatin was performed among paediatric patients with recurrent intracranial and extracranial solid tumours by the Pharmacology Group of the French Pediatric Oncology Society (SFOP) (Vassal *et al*, manuscript in preparation). Finally, the combination of CPT-11 (irinotecan) and TMZ is being studied in phase I trials in adults with recurrent high-grade glioma (Prados, 2001). The rationale of this combination is based on preclinical studies describing a synergistic effect on xenografts models of human glioma and paediatric solid tumours (Houghton *et al*, 2000; Patel *et al*, 2000). Striking are the complete responses that were observed in neuroblastoma, rhabdomyosarcoma and glioblastoma multiforme in these preclinical studies (Houghton *et al*, 2000), making the combination of TMZ and CPT-11 also attractive for clinical trials in paediatric extracranial tumours.

In conclusion, some patients with high-grade glioma may stabilise or respond to a treatment with TMZ, which should be administered >two cycles (unless significant deterioration) since responses may be delayed. Almost 50% of the patients in our series experienced clinical improvement. TMZ is a well-tolerated ambulatory treatment for children with (relapsed) malignant glial tumours and should be combined with other treatment modalities in future clinical trials in CNS tumours.
